# Impact of Human Immunodeficiency Virus on the Burden and Severity of Influenza Illness in Malawian Adults: A Prospective Cohort and Parallel Case-Control Study

**DOI:** 10.1093/cid/cix903

**Published:** 2017-10-16

**Authors:** Antonia Ho, Stephen J Aston, Hannah Jary, Tamara Mitchell, Maaike Alaerts, Mavis Menyere, Jane Mallewa, Mulinda Nyirenda, Dean Everett, Robert S Heyderman, Neil French

**Affiliations:** 1 Institute of Infection and Global Health, University of Liverpool, United Kingdom; 2 Malawi-Liverpool-Wellcome Trust Clinical Research Programme, Blantyre, Malawi; 3 Liverpool School of Tropical of Medicine, United Kingdom; 4 Queen Elizabeth Central Hospital, Blantyre, Malawi; 5 College of Medicine, University of Malawi, Blantyre, Malawi; 6 University College London, United Kingdom

**Keywords:** HIV, influenza, Malawi

## Abstract

**Background:**

The impact of human immunodeficiency virus (HIV) infection on influenza incidence and severity in adults in sub-Saharan Africa is unclear. Seasonal influenza vaccination is recommended for HIV-infected persons in developed settings but is rarely implemented in Africa.

**Methods:**

We conducted a prospective cohort study to compare the incidence of laboratory-confirmed influenza illness between HIV-infected and HIV-uninfected adults in Blantyre, Malawi. In a parallel case-control study, we explored risk factors for severe influenza presentation of severe (hospitalized) lower respiratory tract infection, and mild influenza (influenza-like illness [ILI]).

**Results:**

The cohort study enrolled 608 adults, of whom 360 (59%) were HIV infected. Between April 2013 and March 2015, 24 of 229 ILI episodes (10.5%) in HIV-infected and 5 of 119 (4.2%) in HIV-uninfected adults were positive for influenza by means of polymerase chain reaction (incidence rate, 46.0 vs 14.5 per 1000 person-years; incidence rate ratio, 2.75; 95% confidence interval, 1.02–7.44; *P* = .03; adjusted for age, sex, household crowding, and food security). In the case-control study, influenza was identified in 56 of 518 patients (10.8%) with hospitalized lower respiratory tract infection, and 88 or 642 (13.7%) with ILI. The HIV prevalence was 69.6% and 29.6%, respectively, among influenza-positive case patients and controls. HIV was a significant risk factor for severe influenza (odds ratio, 4.98; 95% confidence interval, 2.09–11.88; *P* < .001; population-attributable fraction, 57%; adjusted for season, sanitation facility, and food security).

**Conclusions:**

HIV is an important risk factor for influenza-associated ILI and severe presentation in this high–HIV prevalence African setting. Targeted influenza vaccination of HIV-infected African adults should be reevaluated, and the optimal mechanism for vaccine introduction in overstretched health systems needs to be determined.

## Teaser

Human immunodeficiency virus (HIV)–related immunosuppression is a major risk factor for influenza illness and severity in Malawian adults. Household crowding, food insecurity, and poor sanitation are additional risk factors. Influenza preventive strategies should target HIV-infected adults in Africa.

Influenza and its complications are leading causes of disease and death worldwide [1]. Vaccination of patient groups at increased risk of influenza-related complications is key to minimizing the impact of disease. Influenza vaccines are currently unavailable in the public sector in most sub-Saharan African countries [2]. The World Health Organization has therefore called for more data on influenza disease burden in the region to guide influenza prevention and control programs [3].

Persons with human immunodeficiency virus (HIV) infection are designated a priority for immunization in many well-resourced countries [4, 5], but data to support this recommendation are inconsistent. HIV does not significantly increase influenza burden or severity in developed settings [6, 7]. Conversely, in low-resource settings with high HIV prevalence there is a higher incidence of influenza illness [8–10] and a greater risk of hospitalization [11–13] and death among HIV-infected persons [9, 14, 15]. Current studies, however, are limited by incomplete ascertainment of HIV status, CD4^+^ cell counts, and antiretroviral treatment (ART), and few have studied the impact of environmental factors, such as household crowding and sanitation. We therefore aimed to determine the impact of HIV on the frequency and severity of adult laboratory-confirmed influenza illness in an urban, high–HIV prevalence African setting, and identify additional risk factors associated with influenza illness and severity.

## METHODS

### Study Setting and Design

Malawi is a low-income Southern African country, with an HIV prevalence of 10.6% [16]. Influenza predominantly circulates between January and April [17]. There is no national influenza immunization program. We performed 2 prospective observational studies at the Queen Elizabeth Central Hospital (QECH), the only inpatient facility providing free healthcare to 1.3 million residents in Blantyre district, and a primary care center adjacent to QECH.

We conducted a cohort study of HIV-infected and HIV-uninfected adults over 2 years; the primary end point was laboratory-confirmed influenza illness. We also performed a case-control study in adults presenting with mild and severe influenza, to establish risk factors for severe influenza (including HIV infection).

## PROCEDURES

### Cohort Study

**Figure 1.**
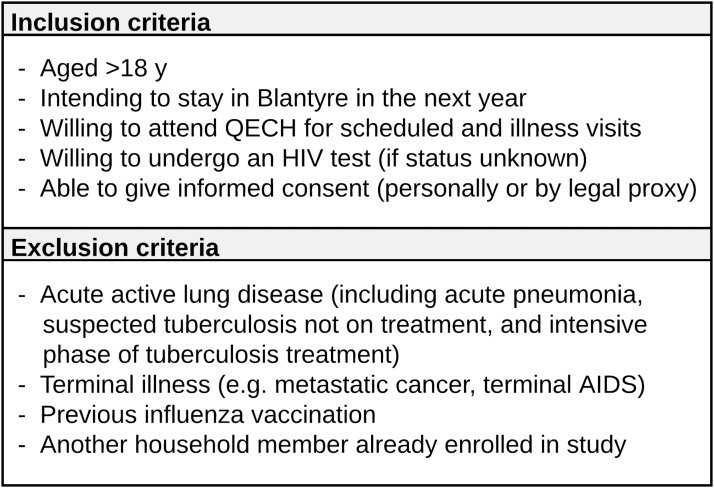
Eligibility criteria for cohort study. Abbreviations: HIV, human immunodeficiency virus; QECH, Queen Elizabeth Central Hospital.

We enrolled adults (aged ≥18 years) from the ART and voluntary counseling and testing clinics at QECH beginning 1 April 2013 (eligibility criteria shown in Figure 1). Active follow-up comprised bimonthly routine clinic reviews. Participants were also instructed to attend the study clinic when they experience an influenza-like illness (ILI), defined as reported or documented fever (≥38°C) and ≥2 of the following symptoms: cough, rhinorrhea, sore throat, myalgia, headache, and vomiting/diarrhea. The study clinician assessed ill participants and instituted appropriate management. Paired nasopharyngeal and oropharyngeal swab samples (FLOQswabs; Copan Diagnostics) were obtained at routine and ILI visits [18].

We compared the incidence of laboratory-confirmed influenza-associated ILI between HIV-infected and HIV-uninfected participants. The at-risk period was calculated from enrollment to the study end date (31 March 2015), death, or loss to follow-up. For participants lost to follow-up, follow-up was censored on the date of relocation, withdrawal of consent, or the last recorded clinic visit.

### Case-Control Study

**Figure 2.**
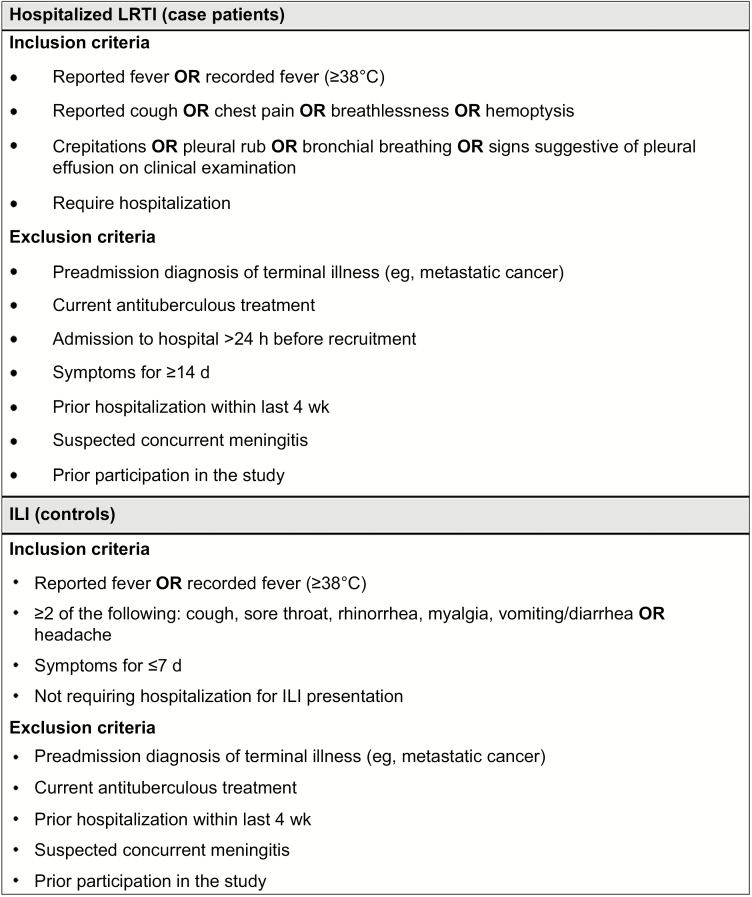
Eligibility criteria for case-control study. Abbreviations: ILI, influenzalike illness; LRTI, lower respiratory tract infection.

Between 15 May 2013 and 28 February 2015, we recruited adults admitted to QECH with acute lower respiratory tract infection (LRTI) (severe cases) and adults attending the primary care center with ILI (nonsevere disease) (eligibility criteria shown in Figure 2). Nasopharyngeal aspirate samples were obtained at enrollment. Participants with influenza-positive hospitalized LRTI and outpatient-managed ILI made up the case patients and controls, respectively.

### Laboratory Procedures

Laboratory testing was performed at the Malawi–Liverpool–Wellcome Trust Clinical Research Programme laboratory. HIV status was established by means of sequential rapid HIV tests (Alere Determine and Uni-Gold, Trinity Biotech) [19]. CD4^+^ cell counts were performed using a FACScount flow cytometer (Becton Dickinson; BD Biosciences). Nasopharyngeal specimens were tested for influenza A and B viruses using the Centers for Disease Control and Prevention human influenza quantitative reverse-transcription polymerase chain reaction (PCR) diagnostic panel and influenza A subtyping kit [20].

### Statistical Analysis

Analysis was performed with Stata software (version 12.1). We tested differences in categorical variables using χ^2^ or Fisher exact test, and differences in continuous variables using *t* or Wilcoxon rank sum test, as appropriate. The cohort study was powered to detect an incidence rate ratio (IRR) of ≥3.0 (α = .05; 2-tailed β = .2), for influenza-associated ILI by HIV status, requiring 608 recruits and allowing for 20% loss-to follow-up, based on an estimated cumulative incidence of 40 per 1000 person-years [PY] in the HIV-uninfected cohort and a 60:40 ratio of HIV infection to noninfection.

Incidence rates of influenza-associated ILI were calculated by dividing the number of events by the number of person-years of follow-up. Poisson regression models were used to estimate IRRs and 95% confidence intervals (CIs) for the effect of HIV and other risk factors on influenza. Age, sex, and HIV infection were included as potential confounders in the multivariable models. Population average Poisson regression models using generalized estimating equations were constructed for recurrent events.

In both studies, stepwise backward elimination of covariates with *P* values < .20 was used to rationalize the multivariable models. We limited the number of covariates in a multivariable model to maintain a limit of 10 events per variable [21]. Two-way interactions were evaluated in all final models. All available case information was used in each univariable analysis. In the multivariable models, we excluded patients with missing data for included variables (data were >95% complete for all variables). The impact of ART and CD4^+^ cell count was assessed in subgroup analyses of HIV-infected individuals.

For the case-control study, a sample size of 57 case patients and 114 unmatched controls provided 80% power to detect an odds ratio (OR) of ≥2.5. Based on observed influenza prevalences of 11% and 16.4% in adults presenting to QECH with severe or mild acute respiratory illness, respectively, in our sentinel surveillance (unpublished data), we estimated recruitment of approximately 518 adults with hospitalized LRTI and 695 adults with ILI.

We estimated the OR of having HIV infection and other potential risk factors for severe influenza in case patients and controls, controlling for confounders, using unconditional logistic regression models. HIV status and recruitment season were included a priori in the multivariable model. Population-attributable fractions of modifiable risk factors for severe influenza were estimated from the prevalence of exposure in case patients and adjusted OR from the multivariable logistic regression model [22]. Ethical approval was provided by the University of Malawi College of Medicine Research Ethics Committee (Study No. P.11/12/1310) and the Research Ethics Committee of the University of Liverpool (Study No. 12.43).

## RESULTS

### Cohort Study: Participant Characteristics

**Figure 3.**
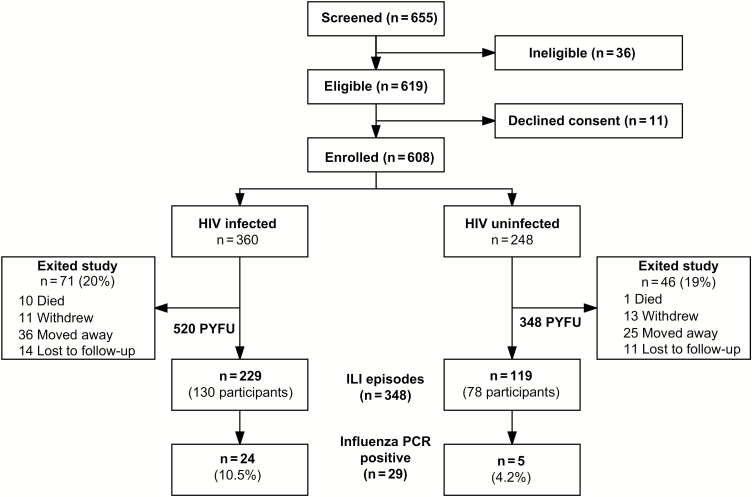
Recruitment and progress of cohort participants. Flow diagram for recruitment, loss to follow-up, and influenza-like illness events among cohort participants. Reasons for ineligibility included intention to relocate out of Blantyre (n = 10), inability to attend regular study visits (n = 6), inability to give written informed consent (n = 1), evidence of active acute respiratory disease at enrollment (n = 17), and enrollment of another household member in the study (n = 2). Reasons for declining consent included not having time (n = 3), not interested (n = 2), fear of participation (n = 4), and wished to seek spouse’s consent (n = 2). Abbreviations: HIV, human immunodeficiency virus; ILI, influenzalike illness; PCR, polymerase chain reaction; PYFU, person-years follow-up.

In total, 608 adults were enrolled; 360 (59%) had HIV infection (Figure 3 and Table 1). Compared with HIV-uninfected participants, HIV-infected participants were older (median age, 37 vs 31 years; *P* < .001), and a higher proportion were female (69% vs 55%; *P* = .001). Chronic lung disease and smoking were uncommon in both groups. A significantly higher proportion of HIV-infected participants reported previous tuberculosis (25% vs 2%; *P* < .001) and pneumonia in the past 5 years (16% vs 5%; *P* < .001).

**Table 1.** Demographic, Clinical, Household, and Socioeconomic Characteristics of the Cohort Participants

Characteristic	Participants, No./Total (%)^a^		*P* Value for Difference^b^
	HIV Infected (n = 360)	HIV Uninfected (n = 248)	
Male sex	113/360 (31)	111/248 (45)	.001
Age, median (IQR), y	37 (31–45)	31 (25–39)	<.001^c^
Medical history			
Asthma	18/360 (5)	8/248 (3)	.29
Chronic lung disease	1/360 (0.3)	0/248 (0)	1.0
Chronic cardiac disease	2/360 (0.6)	1/248 (0.4)	.80
Chronic kidney disease	0/360 (0)	0/248 (0)	…^d^
Chronic liver disease	2/359 (0.6)	0/248 (0)	.24
Pregnant (at enrollment)	4/359 (1)	4/248 (2)	.84
Previous pulmonary tuberculosis	90/359 (25)	6/248 (2)	<.001
Pneumonia in past 5 y	59/358 (16)	13//248 (5)	<.001
Current smoker	11/360 (3)	4/248 (2)	.14^e^
Drinks alcohol	44/359 (12)	35/248 (14)	.47
Body mass index <18.5 kg/m^2^	38/346 (11)	17/242 (7)	.18
Household and socioeconomic factors			
No. of children aged <5 y in household			
0	219/358 (61)	132/247 (54)	
1	114/358 (32)	83/247 (33)	
≥2	25/358 (7)	32/247 (13)	.03
No. of individuals aged >5 y in household			
0–2	89/358 (25)	56/247 (23)	
3–4	161/358 (45)	91/247 (37)	
≥5	108/358 (30)	100/247 (41)	.03
Crowding index^f^			
<1.5	110/360 (30)	66/248 (27)	
1.5–2.4	144/360 (40)	95/248 (38)	
>2.5	106/360 (30)	87/248 (35)	.33
Sanitation facility			
None/non-VIP toilet	327/359 (91)	205/248 (83)	
VIP toilet/flush toilet	32/359 (9)	43/248 (17)	.002
Water supply			
River/stream/borehole	75/354 (21)	44/246 (18)	
Public tap/standpipe	212/354 (60)	131/246 (53)	
Piped to dwelling	67/354 (19)	71/246 (29)	.02
Principal cooking fuel/energy source			
Firewood	77/357 (22)	44/246 (18)	
Charcoal	244/357 (68)	131246 (53)	
Electricity	36/357 (10)	71/246 (29)	.66
Highest level of education			
Never attended school	18/359 (5)	10/248 (4)	
Primary	152/359 (42)	89/248 (36)	
Secondary/tertiary	189/359 (53)	149/248 (60)	.19
Unemployed	87/359 (24)	47/248 (19)	.13
Assets owned^g^			
1–2	141/360 (39)	83/248 (33)	
3	151/360 (42)	113/248 (46)	
4–5	68/360 (19)	52/248 (21)	.36
Difficulties obtaining food			
Never	137/356 (38)	133/247 (54)	
1–2 times/mo	149/356 (42)	86/247 (35)	
>2 times/mo	70/356 (20)	28/247 (11)	<.001

Abbreviations: HIV, human immunodeficiency virus; IQR, interquartile range; VIP, ventilated improved pit latrine.

^a^Data represent No./total (%) of participants unless otherwise specified.

^b^Determined with Mantel-Haenszel χ^2^ test unless stated otherwise.

^c^Wilcoxon rank sum test.

^d^For Chronic kidney disease: Chi-square or Fisher’s exact test not possible when neither group has the exposure of interest.

^e^Fisher exact test.

^f^The crowding index was calculated as the number of persons in the household divided by the number of sleeping rooms.

^g^Number of the following assets owned in household: working refrigerator, radio, mobile phone, bed, and car or motorbike.

HIV-uninfected participants had larger households, but crowding was similar in the 2 groups. HIV-uninfected participants had better sanitation facilities, water supply, and food security. No significant differences were observed in education level, employment, or asset ownership.

Of the 360 HIV-infected participants, 234 (65.0%) were receiving ART at enrolment, the majority (82%) for >12 months. The median CD4^+^ cell count at enrollment was 390/µL (interquartile range, 244–547/µL).

Eleven participants died during the study (HIV infected, n = 10); none had reported respiratory symptoms before death. Sixty-one participants (10%) migrated out of Blantyre, 24 (3.9%) withdrew consent, and 25 (4.1%) were lost to follow-up (Figure 3). There was no differential loss to follow-up by HIV status. The total person-time follow-up was 520 and 348 PY in the HIV-infected and HIV-uninfected cohorts, respectively.

### Impact of HIV Infection on Influenza-Associated ILI Incidence

We recorded 348 ILI episodes in 208 participants (HIV infected, n = 130) (clinical characteristics show in Supplementary Table S1). The incidences of ILI presentation were 442 and 341 per 1000 PY in the HIV-infected and HIV-uninfected participants, respectively (IRR, 1.21; 95% CI, .99–1.48). Twenty-nine ILI episodes (8.3%) had PCR results positive for influenza, including influenza A(H3N2) (n = 11) and influenza B (n = 18) and occurring in 24 (10.5%) of 229 HIV-infected and 5 (4.2%) of 119 HIV-uninfected participants. The incidence of laboratory-confirmed influenza-associated ILI per 1000 PY was 46.0 (95% CI, 30.8–68.6) in HIV-infected and 14.5 (6.0–34.7) in HIV-uninfected adults (IRR, 3.21; 95% CI, 1.22–8.41) (Table 2).

**Table 2.** Risk Factors for Laboratory-confirmed Influenza Illness Among Cohort Participants

Characteristic	Incidence Rate per 1000 PY of Follow-up (95% CI)	Univariable Analysis^a^		Multivariable Analysis^a,b^	
		IRR (95% CI)	*P* Value	IRR (95% CI)	*P* Value
Sex					
Male	31.4 (16.9–58.3)	1	…	1	…
Female	34.6 (22.1–54.2)	1.21 (0.52–2.84)	.66	0.88 (0.40–1.93)	.74
Age group, y					
18–29	20.9 (8.7–50.2)	1	…	1	…
30–39	39.9 (23.2–68.7)	1.73 (0.60–4.97)	…	1.55 (0.54–4.43)	…
≥40	36.3 (20.1–65.6)	1.33 (0.43–4.05)	.30	1.42 (0.47–4.28)	.70
HIV status					
Uninfected	14.5 (6.0–34.7)	1	…	1	…
Infected	46.0 (30.8–68.6)	3.21 (1.22–8.41)	.02	2.75 (1.02–7.44)	.03
Medical history					
Previous pulmonary tuberculosis	28.2 (23.3–51.1)	0.82 (0.28–2.43)	.87	…	…
Pneumonia in past 5 y	54.3 (24.4–120.9)	1.86 (0.75–4.57)	.14	…	…
Body mass index <18.5 kg/m^2^	40.3 (13.0–125.0)	0.81 (0.25–2.68)	.73	…	…
Housing characteristics					
No. of children aged <5 y in household			…	…	…
0	36.2 (22.8–57.5)	1	…		…
1	27.8 (13.9–55.6)	0.77 (0.33–1.77)			…
≥2	37.3 (12.0–115.5)	1.03 (0.30–3.50)	.80		…
No. of individuals aged ≥5 y in household			…	…	…
0–2	25.5 (10.6–61.3)	1	…		…
3–4	28.1 (15.1–52.3)	1.10 (0.38–3.22)	…		…
≥5	44.7 (26.5–75.4)	1.76 (0.63–4.87)	.41		…
Crowding index^c^					
<1.5	16.3 (6.12–43.5)	1	…	1	…
1.5–2.4	47.1 (28.8–76.8)	2.88 (0.96–8.62)	…	3.41 (1.12–10.36)	…
≥2.5	32.0 (16.6–61.5)	1.95 (0.60–6.35)	.11	2.06 (0.62–6.83)	.06
Socioeconomic characteristics					
Highest level of education			…	…	…
Never attended/primary	47.6 (30.0–75.6)	1	…		…
Secondary/tertiary	22.5 (12.5–40.7)	0.47 (0.22–1.00)	.05		…
Employment			…	…	…
No	49.6 (28.8–85.4)	1	…		…
Yes	26.4 (16.2–43.1)	0.53 (0.26–1.11)	.09		…
Food security: difficulty obtaining food					
Never	25.9 (13.9–48.2)	1	…	1	…
1–2 times/mo	20.7 (9.9–43.5)	0.80 (0.30–2.10)	…	0.71 (0.27–1.88)	…
>2 times/mo	87.2 (49.5–153.6)	3.35 (1.45–7.76)	.005	3.09 (1.30–7.36)	.006

Abbreviations: CI, confidence interval; HIV, human immunodeficiency virus; IRR, incidence rate ratio; PY, person-years.

^a^IRRs estimated for the incidence of laboratory-confirmed influenza, using Poisson regression.

^b^Adjusted for sex, age group, HIV status, crowding index, and food security.

^c^The crowding index was calculated as the number of persons in the household divided by the number of sleeping rooms.

In the univariable analysis, history of previous pneumonia, household crowding, lower education level, unemployment, and food insecurity were associated with influenza-related ILI (Table 2). After adjustment for age, sex, household crowding and food security, HIV-infected adults had an approximately 3-fold increased rate of influenza-associated ILI compared with HIV-uninfected adults (adjusted IRR [aIRR], 2.75; 95% CI, 1.02–7.44; Pearson’s goodness-of-fit test, *P* = .12).

Among HIV-infected participants, individuals with enrollment CD4^+^ cell counts <200/µL had a higher incidence of influenza-associated ILI than those with counts >200/µL, but the association was nonsignificant (79.1 vs 40.5 per 1000 PY; IRR, 1.95; 95% CI, .78–4.92). The effect of HIV on influenza did not differ by ART status.

### Other Risk Factors for Influenza Infection

A household crowding index (number of persons in the household divided by the number of sleeping rooms) of 1.5–2.4 was associated with a 3-fold increased risk of influenza-associated ILI, compared with households with <1.5 persons per sleeping room (aIRR, 3.41; 95% CI, 1.12–10.36). The increased risk was not observed in participants who lived in household with a crowding index >2.5 (2.06; .62–6.83) (Table 2). Participants who reported difficulties accessing food more than twice per month had a 3-fold increased risk of influenza-associated ILI, compared with those with no food access difficulties (aIRR, 3.09; 95% CI, 1.30–7.36). Food insecurity was also a possible effect modifier of the impact of HIV infection on influenza (*P* = .01). Among those with frequent difficulty obtaining food, HIV infection was associated with 10-fold increased incidence of influenza-associated ILI (IRR, 9.50; 95% CI, 1.27–70.99), but no association was evident in those with little or no food insecurity (IRR, 1.18; 95% CI, .32–4.39). No interaction was demonstrated with the other covariates.

### Case-Control Study: Patient Characteristics

**Figure 4.**
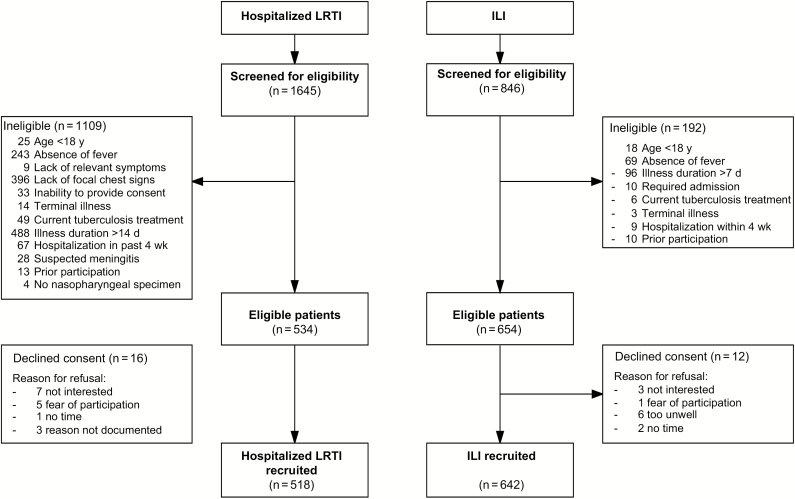
Case-control study recruitment. Abbreviations: ILI, influenza-like illness; LRTI, lower respiratory tract infection.

A total of 1645 patients were assessed for eligibility for hospitalized LRTI and 846 for ILI, with subsequent recruitment of 518 and 642 patients, respectively (Figure 4). Patients with severe respiratory presentation were older (median age, 35 vs 32 years; *P* < .001), predominantly male (62% vs 43%; *P* < .001), and had a substantially higher prevalence of HIV infection (77% vs 30%; *P* < .001) (Table 3). They also had a higher prevalence of previous pulmonary tuberculosis (18% vs 7%), pneumonia within the past 5 years (22% vs 7%), smoking (11% vs 6%), and regular alcohol intake (26% vs 12%) (all *P* < .001). Sanitation facility, water supply, cooking fuel, asset ownership, education level, and food security were also worse among patients with hospitalized LRTI.

**Table 3.** Demographic, Clinical, Household, and Socioeconomic Characteristics of Adults Enrolled With Hospitalized Lower Respiratory Tract Infection and Mild Influenza-like Illness

Characteristic	Participants, No./Total (%)^a^		*P* Value for Difference^b^
	Hospitalized LRTI (n = 518)	ILI (n = 642)	
Male sex	323/518 (62)	273/642 (43)	<.001
Age, median (IQR), y	35 (30–42)	32 (25–43)	<.001^c^
Prior treatment			
Attended another health facility^d^	312/513 (61)	62/635 (10)	<.001
Antibiotics within 2 wk^e^	309/509 (61)	70/630 (11)	<.001
Antimalarials within 2 wk	83/511 (16)	13/630 (2)	<.001
HIV status			
HIV infected	396/517 (77)	192/640 (30)	<.001
CD4^+^ cell count, median (IQR) cells/µL	101 (46–196)	313 (167–450)	<.001^c^
Receiving ART at enrollment^f^	188/233 (81)	68/87 (78)	.62
Medical history			
Chronic lung disease	18/503 (4)	15/635 (2)	.23
Chronic cardiac disease	2/503 (0.4)	1/635 (0.2)	.59^g^
Hypertension	9/503 (2)	19/635 (3)	.19
Chronic kidney disease	1/503 (0.2)	0/503 (0)	.44^g^
Chronic liver disease	1/503 (0.2)	0/503 (0)	.44^g^
Pregnant	2/195 (0)	10/369 (3)	.19^g^
Previous pulmonary tuberculosis	90/514 (18)	45/638 (7)	<.001
Pneumonia in past 5 y	113/512 (22)	46/638 (7)	<.001
Body mass index <18.5 kg/m^2^	147/495 (30)	67/638 (11)	<.001
Current smoker	58/513 (11)	39/638 (6)	<.001
Drinks alcohol	135/512 (26)	78/638 (12)	<.001
Household and socioeconomic factors			
No. of children aged <5 y in household			
0	296/513 (58)	381/638 (60)	
1	156/513 (30)	195/638 (30)	
≥2	61/513 (12)	62/638 (10)	.48
Crowding index^h^			
<1.5	174/497 (35)	219/612 (36)	
1.5–2.4	203/497 (41)	258/612 (42)	
≥2.5	120/497 (24)	135/612 (22)	.71
Sanitation facility			
None/non-VIP toilet	284/514 (55)	260/637 (41)	
VIP toilet	188/514 (37)	252/637 (39)	
Flush toilet	42/514 (8)	125/637 (20)	<.001
Water supply			
River/stream/borehole	150/514 (29)	134/638 (21)	
Public tap/standpipe	272/514 (53)	308/638 (48)	
Piped to dwelling	92/514 (18)	196/638 (31)	<.001
Principal cooking fuel/energy source			
Firewood	140/514 (27)	129/637 (20)	
Charcoal	325/514 (63)	421/637 (66)	
Electricity	49/514 (10)	87/637 (14)	.006
Highest level of education			
Never attended school	49/508 (10)	52/638 (8)	
Primary	274/508 (54)	266/638 (42)	
Secondary/tertiary	185/508 (36)	320/638 (50)	<.001
Unemployed	68/514 (13)	91/638 (14)	.61
Assets owned^i^			
0	84/515 (16)	75/638 (12)	
1	106/515 (20)	112/638 (18)	
2	122/515 (24)	165/638 (26)	
3	158/515 (31)	206/638 (32)	
4–5	35/515 (9)	80/638 (12)	.04
Difficulties obtaining food			
Never	247/514 (48)	367/638 (58)	
1–2 times/mo	226/514 (44)	250/638 (39)	
>2 times/mo	41/514 (8)	31/638 (3)	<.001
Season of recruitment			
January–March	99/518 (19)	204/642 (32)	
April–June	100/518 (19)	180/642 (28)	
July–September	161/518 (31)	145/642 (24)	
October–December	15/518 (31)	102/642 (16)	<.001

Abbreviations: ART, antiretroviral treatment; HIV, human immunodeficiency virus; ILI, influenzalike illness; IQR, interquartile range; LRTI, lower respiratory tract infection, VIP, ventilated improved pit latrine.

^a^Data represent No./total (%) unless otherwise specified.

^b^Determined with Mantel-Haenszel χ^2^ test unless stated otherwise.

^c^Wilcoxon rank sum test.

^d^Including attendance at another hospital, health center, private clinic, traditional healer, or pharmacy.

^e^Excluding cotrimoxazole prophylaxis in HIV-infected individuals.

^f^Denominator is the number of patients with known HIV infection at enrollment.

^g^Fisher exact test.

^h^The crowding index was calculated by the number of persons in the household divided by the number of sleeping rooms.

^i^Number of the following assets owned in household: working refrigerator, radio, mobile phone, bed, and car/motorbike.

### Contribution of Influenza to Mild and Severe Respiratory Infection

Influenza was identified in 56 (10.8%) patients with hospitalized LRTI (case patients) and 88 (13.7%) patients with ILI (controls) (Table 4). Influenza A virus was detected in 30 (53.6%) of the case patients and 35 (39.8%) of the controls; all had influenza A(H3N2) except 1 control (unsubtyped). Two controls were positive for both influenza A(H3N2) and B. Influenza A(H1N1)pdm09 was not detected during the study period.

**Table 4.** Risk Factors for Severe Influenza Presentation in Influenza-Positive Case Patients and Controls

Characteristic	Participants, No./Total (%)		Univariable Analysis^c^		Multivariable Analysis^c,d^	
	Case Patients (n = 56)^a^	Controls (n = 88)^b^	OR (95% CI)	*P* Value	OR (95% CI)	*P* Value
Male sex	28/56 (50)	43/88 (49)	0.96 (0.49–1.88)	.89	…	…
Age group, y				…	…	…
18–29	17/56 (30)	36/88 (41)	0.75 (0.32–1.73)	.32		…
30–39	22/56 (39)	25/88 (28)	1.40 (0.61–3.22)	…		…
≥40	17/56 (30)	27/88 (31)	1	…		…
HIV infected	39/56 (70)	26/88 (30)	5.47 (2.63–11.36)	<.001	4.98 (2.09–11.88)	<.001
Medical history				…		
Previous pulmonary tuberculosis	10/56 (18)	5/88 (6)	3.61 (1.16–11.20)	.03	…	…
Pneumonia in past 5 y	17/55 (31)	7/88 (8)	5.18 (1.98–13.53)	.001	6.49 (1.95–21.25)	.001
Body mass index <18.5 kg/m^2^	12/53 (23)	9/88 (10)	2.57 (1.00–6.60)	.05		…
Housing and socioeconomic characteristics						
Sanitation facility						
None/non-VIP toilet	34/55 (61)	37/88 (42)	2.12 (1.08–4.22)	.03	3.14 (1.25–7.84)	.01
VIP toilet/flush toilet	22/55 (40)	51/88 (58)	1	…	1	…
Water supply				…	…	…
River/stream/borehole	15/56 (27)	19/88 (22)	2.54 (0.93–6.98)	.07		…
Public tap/standpipe	32/56 (57)	40/88 (45)	2.58 (1.07–6.22)	…		…
Piped to dwelling	9/56 (16)	29/88 (33)	1	…		…
Highest level of education				…	…	…
Never attended	5/55 (9)	15/88 (17)	1	…		…
Primary	34/55 (62)	28/88 (32)	3.64 (1.18–11.27)	…		…
Secondary/tertiary	16/55 (29)	45/88 (51)	1.07 (0.33–3.41)	.002		…
Difficulties obtaining food						
Never	28/56 (50)	58/88 (66)	1	…	1	…
1–2 times/mo	21/56 (38)	29/88 (33)	1.50 (0.73–3.08)	…	1.15 (0.47–2.84)	…
>2 times/mo	7/56 (12)	1/88 (1)	14.4 (1.70–123.65)	.007	20.85 (1.97–221.16)	.01
Month of recruitment						
January–March	16/56 (29)	49/88 (56)	1	…	1	…
April–June	15/56 (27)	18/88 (20)	2.55 (1.05–5.20)	…	3.36 (1.13–9.95)	…
July–September	12/56 (21)	9/88 (10)	4.08 (1.45–11.46)	…	6.34 (1.69–23.80)	…
October–December	13/56 (23)	12/88 (14)	3.32 (1.25–8.72)	.01	3.60 (1.07–12.10)	.01

Abbreviations: CI, confidence interval; HIV, human immunodeficiency virus; OR, odds ratio; PCR, polymerase chain reaction; VIP, ventilated improved pit latrine.

^a^Case had severe (hospitalized) lower respiratory tract infection and a positive PCR result for influenza.

^b^Controls had influenza-like illness and a positive PCR result for influenza.

^c^Unconditional logistic regression.

^d^Adjusted for HIV status, history of pneumonia within 5 years, month of recruitment, sanitation facility, and food security.

### Risk Factors for Severe Influenza Presentation

Among the 56 case patients and 88 controls, no difference in age and sex was observed (Table 4). Thirty nine (69.6%) of the case patients and 26 (29.6%) of the controls were HIV-infected (*P* < .001). Compared with HIV-infected controls, HIV-infected case patients had more advanced immunosuppression (median CD4^+^ cell count, 140/µL vs 265/µL; *P* = .03) and were more likely to be receiving ART (35.7 vs 9.1%; *P* < .001). Case patients were more likely to have a low body mass index and report a history of tuberculosis and pneumonia. Controls had better sanitation, education, and food security. However, household exposure to children <5 years old or crowding did not differ according to case-control status.

In the univariable analysis, HIV infection, reported history of tuberculosis, pneumonia within past 5 years, low body mass index (<18.5 kg/m^2^), month of recruitment, type of water supply, sanitation facility, lower education level, and food insecurity were associated with being a case patient (Table 4). Month of recruitment was included in the multivariable model a priori owing to seasonal discrepancy in the recruitment of patients with hospitalized LRTI or ILI. In the multivariable model, HIV infection was strongly associated with severe influenza (adjusted OR, 4.98; 95% CI, 2.09–11.88; *P* < .001). In addition, reported pneumonia within 5 years (adjusted OR, 6.49; 95% CI, 2.00–21.07; *P* < .001), poor sanitation facility (3.14; 1.25–7.84; *P* = .01); and frequent difficulty accessing food (20.85; 1.97–221.16; *P* = .01) were independent risk factors for severe influenza.

In a subgroup analysis of HIV-infected case patients and controls, there was a trend toward lower CD4^+^ cell counts in case patients (OR, 2.72; 95% CI, .97–7.60; CD4^+^ cell count, <200/µL vs >200/µL). No association was found between ART status and influenza severity.

### Population-Attributable Fraction

The highest proportions of hospitalized influenza cases were attributed to HIV infection (adjusted population-attributable fraction, 56.7%; 95% CI, 42.3–67.4) and poor sanitation (40.9%; 20.5–56.0) (Table 5). Frequent difficulty accessing food accounted for 12% (95% CI, 10.6–13.6) of hospitalized influenza cases.

**Table 5.** Population-Attributable Fraction of Modifiable Risk Factors for Severe Influenza

Risk Factor	Proportion of Case Patients Exposed, %	Adjusted OR (95% CI)	*P* Value	Adjusted Population-Attributable Fraction (95% CI), %
HIV infection	70	4.98 (2.09–11.88)	<.001	56.7 (42.3–67.4)
Sanitation facility				
None/non-VIP toilet	39	3.14 (1.25–7.84)	.01	40.9 (20.5–56.0)
VIP/flush toilet	61	1		
Difficulties obtaining food				
Never	50	1		…
1**–**2 times/wk	38	1.15 (0.47–2.84)		6 (−40.9 to 38.0)
>2 times/wk	12	20.85 (1.97–221.16)	.01	12.0 (10.3–13.7)

Abbreviations: CI, confidence interval; HIV, human immunodeficiency virus; OR, odds ratio; VIP, ventilated improved pit latrine.

## DISCUSSION

In this urban African adult population, HIV infection is an important risk factor for both symptomatic influenza and severe illness. Compared with HIV-uninfected adults, HIV-infected adults had a 3 times higher incidence of influenza-associated ILI, and a 5-fold greater odds of severe influenza disease. Furthermore, nearly 60% of influenza-related hospitalized LRTI cases were attributable to HIV. Although neither study was powered to examine the effect of HIV at different levels of immunosuppression, our data suggest higher incidence and greater disease severity among those with CD4^+^ cell counts <200/µL.

Previous population-level surveillance and retrospective studies in South Africa and Kenya that have examined the association between HIV and influenza found a higher disease burden [8–10] and an increased risk of influenza-associated hospitalization in HIV-infected persons [11–13], particular among those with severe immunosuppression [13]. The current studies provide robust evidence to support these findings having prospectively ascertained HIV status, CD4^+^ cell count, and information on ART, as well as other exposures, including household and socioeconomic characteristics that may confound the association between HIV and influenza.

We found a higher incidence of influenza-associated ILI among HIV-infected than among HIV-uninfected individuals. Although this could indicate an increased susceptibility to infection, it may instead, or also, reflect a greater propensity of HIV-infected individuals to develop symptomatic illness after influenza infection, which has been described elsewhere [23]. This is a pertinent finding in high–HIV prevalence settings, because HIV-infected individuals may play an important role in the community transmission of influenza.

Taken together, data from the 2 studies present a persuasive argument for a strong association between HIV and influenza. The finding that more than half of hospitalized influenza presentations in Malawian adults were attributable to HIV further emphasizes its critical role in severe influenza in this population. Because pneumonia is the most common cause of adult medical admissions at QECH [24], effective influenza preventive strategies could substantially reduce the burden of acute respiratory infections in Malawi and other similar resource-limited settings.

Inactivated influenza vaccines have demonstrated safety and efficacy in HIV-infected persons [25]. Clinical efficacy of 75.5% has been reported in South African HIV-infected adults, but the trial excluded patients with comorbid conditions and ART-naive patients with CD4^+^ cell counts <100/μL [26]. Influenza vaccines are not widely deployed in most African countries [2, 27]. Before consideration of HIV-infected persons as a target group for immunization, policy makers will require evidence of vaccine efficacy in the context of advanced immunosuppression and/or comorbid conditions, as well as the potential public health impact and cost-effectiveness of vaccinating HIV-infected individuals, compared with other target groups (eg, pregnant women, young children). The acceptability of annual vaccination, feasibility of vaccine administration at ART clinics, and optimal timing of vaccination in the absence of clear seasonality will require elucidation. Numerous regulatory, logistical, and financial obstacles will need to be overcome if targeted influenza vaccination policies are to be successfully and sustainably implemented in the region.

The introduction of ART was associated with a dramatic decline in influenza-attributable hospitalizations in the United States [28], and improved survival after pandemic influenza A(H1N1) infection in Mexico [29]. However, this beneficial effect has not been observed in Africa. Cohen et al [30] found no difference in case fatality ratio by ART status among HIV-infected individuals with influenza-positive severe acute respiratory illness [30]. A Malawian study demonstrated poor reconstitution of influenza-specific CD4^+^ T-cell response in HIV-infected adults after 12 months of highly active ART, despite a rise in CD4^+^ cell count [31]. Hence the impact of ART on the relationship between HIV and influenza severity requires further evaluation.

Identification of household crowding, poor sanitation, and food insecurity as risk factors for influenza highlight the importance of current public health interventions to alleviate hunger and poverty and improve access to clean water and sanitation [32]. Food insecurity emerged as a previously unrecognized risk factor for both influenza illness and severity. More in-depth evaluation of food insecurity and its association with influenza and other respiratory infections is warranted [33].

Our results, along with other data from the region, contrast with those from developed settings [6]. This highlights the limitations of extrapolating findings from developed settings to inform influenza control policies in Africa. Differences in the observed impact of HIV on influenza burden and severity may be due to more advanced immunosuppression, poorer access to ART, higher prevalence of comorbid conditions, poverty-related factors identified in this study, compared with other regions.

Our study has several limitations. First, it was conducted in a single urban center, which may limit the generalizability of our findings to rural populations. Second, this study was not powered to assess the impact of CD4^+^ cell count or ART on influenza incidence and severity. CD4^+^ cell counts were measured during acute illness in the case-control study; the degree of immunosuppression may not be accurately represented because CD4^+^ cell count depression can occur during acute illness. Third, passive surveillance was used for case ascertainment in both studies; underascertainment of ILI episodes, and therefore influenza cases, is conceivable. Biased estimates away from the null could have resulted if HIV-infected cohort participants were more likely than uninfected individuals to present with ILI . Similar bias could have arisen if HIV-infected individuals had a higher propensity to attend hospitals with severe respiratory symptoms in the case-control study. However, there was no significant difference in the incidence of ILI between HIV-infected and uninfected cohorts, and a greater proportion of HIV-related ILI cases had severe clinical signs (Supplementary Table S1). Furthermore, the majority of case-control study participants were unaware of their HIV status at enrollment. Finally, we were unable to control for bacterial coinfection in the case-control study, because diagnostic tests for bacteria were undertaken in case patients but not controls.

We have comprehensively evaluated the association between HIV and influenza, identifying HIV-infected persons at particular risk of symptomatic influenza and severe disease. Influenza-preventive strategies should be an important aspect of the management of HIV-infected adults in sub-Saharan Africa. Further studies are needed in Malawi and other high–HIV prevalence settings to determine influenza vaccine efficacy in persons with advanced immunosuppression and evaluate the potential public health impact ahead of operational research addressing the logistical barriers to implementing large-scale vaccination programs.

## Supplementary Data

Supplementary materials are available at *Clinical Infectious Diseases* online. Consisting of data provided by the authors to benefit the reader, the posted materials are not copyedited and are the sole responsibility of the authors, so questions or comments should be addressed to the corresponding author.

Supplementary Data

## Notes

***Acknowledgments.*** Marc-Alain Widdowson and Meredith McMorrow (US Centers for Disease Control and Prevention) provided input into study implementation. Nico Nagelkerke (Malawi–Liverpool–Wellcome Trust Clinical Research Programme) provided statistical analysis support.

***Financial support.*** This work was supported by the Wellcome Trust (grant 097464/Z/11/A).

***Potential conflicts of interest.*** N. F. has received grant support from GlaxoSmithKine outside the submitted work. All other authors report no potential conflicts. All authors have submitted the ICMJE Form for Disclosure of Potential Conflicts of Interest. Conflicts that the editors consider relevant to the content of the manuscript have been disclosed.
