# Supplementary material for: Impact of Human Immunodeficiency Virus on the Burden and Severity of Influenza Illness in Malawian Adults: A Prospective Cohort and Parallel Case-Control Study
Source: Clin Infect Dis. 2017 Oct 16;66(6):865–76. doi: 10.1093/cid/cix903 (PMC5850564; doi:10.1093/cid/cix903)
Supplement: Supplementary Data [file cix903_suppl_supplementary_data.docx]

**SUPPLEMENTARY DATA**

**Supplementary table 1. Cohort study: Clinical characteristics of ILI episodes**

| Characteristic | HIV-infected participants | HIV-uninfected participants |
| --- | --- | --- |
|  | **(N=229)** | **(N=119)** |
| Symptoms |  |  |
| Cough | 167/229 (73) | 85/119 (72) |
| Sore throat | 123/229 (54) | 59/119 (50) |
| Rhinorrhoea | 200/229 (87) | 102/119 (86) |
| Myalgia | 191/229 (83) | 100/119 (85) |
| Nausea/vomiting | 25/229 (11) | 10/119 (8) |
| Diarrhoea | 36/229 (16) | 10/119 (8) |
| Shortness of breath | 26/229 (11) | 5/119 (4) |
| Headache | 196/229 (86) | 102/119 (86) |
| Clinical signs |  |  |
| Temperature >38°c | 21/229 (9) | 2/119 (2) |
| Heart rate >120 beats/min | 11/229 (5) | 0/119 (0) |
| Respiratory rate >30/min | 5/229 (2) | 0/119 (0) |
| Oxygen saturation <90% | 3/229 (1) | 0/119 (0) |
| Upper respiratory tract signs | 29/229 (13) | 6/119 (5) |
| Lower respiratory tract signs | 5/229 (2) | 1/119 (1) |
| Hospital admission | 5/229 (2) | 0/119 (0) |
| Data are n/N (%). | | |
